# Editorial: Safety and side effects of psychotropic medications, volume II

**DOI:** 10.3389/fpsyt.2023.1326118

**Published:** 2023-12-06

**Authors:** Renato de Filippis, Mireia Solerdelcoll, Mohammadreza Shalbafan

**Affiliations:** ^1^Psychiatry Unit, Department of Health Sciences, University Magna Graecia of Catanzaro, Catanzaro, Italy; ^2^Department of Child and Adolescent Psychiatry, Institute of Psychiatry, Psychology and Neuroscience, King's College London, London, United Kingdom; ^3^Department of Child and Adolescent Psychiatry and Psychology, Institute of Neuroscience, Hospital Clínic de Barcelona, Barcelona, Spain; ^4^Department of Medicine, University of Barcelona, Barcelona, Spain; ^5^Mental Health Research Center, Psychosocial Health Research Institute (PHRI), Department of Psychiatry, School of Medicine, Iran University of Medical Sciences, Tehran, Iran

**Keywords:** adverse drug reaction (ADR), adverse effects, mental disorders, mental health, psychiatry, psychopharmacology, safety, tolerability

The safety and tolerability of psychotropic medications have always been a significant concern within the realm of mental health treatment (1, 2). Over recent years, there has been a noticeable uptick in the collection of evidence-based data and dedicated research endeavors aimed at gaining a more comprehensive understanding of the advantages and drawbacks tied to these medications (3, 4). This surge in empirical research has empowered healthcare providers to make more informed decisions when prescribing psychotropic drugs and personalizing treatment plans to meet each patient's unique needs (5, 6). Furthermore, research has been actively engaged in a collaborative effort to devise new strategies and medications boasting enhanced safety profiles and reduced adverse drug reactions (ADRs) (7, 8). As the field continually evolves, the focus on patient safety and the fine-tuning of psychotropic treatments remains an overarching priority, with the ultimate goal of improving mental health outcomes for individuals worldwide (9, 10).

Therefore, following the first volume of this Research Topic (11), our objective was to compile articles that delve into the safety and ADRs of psychotropic medications. We invited contributions from all sources that could potentially provide fresh insights and evidence to this critical subject matter.

Akbarzadeh et al. evaluated the effect of adding an extract of the plant Alpinia galanga, known for enhancing sexual function, to the treatment of adult males taking selective serotonin reuptake inhibitors (SSRIs) with the goal to improve SSRI-induced erectile dysfunction. They designed a triple-blind randomized clinical trial involving 60 adult male participants on SSRIs, receiving 500 mg of Alpinia galanga extract or placebo, and adding Alpinia galanga to the SSRIs treatment seems to be a promising approach to alleviate the SSRI-related sexual dysfunction in male.

Kimura et al. conducted a systematic review aimed to assess the heart safety of escitalopram in comparison to a placebo for patients with underlying cardiovascular issues (12). The analysis included five randomized controlled trials with a total of 773 participants. Escitalopram did not significantly increase the risk of major adverse cardiovascular events, medication discontinuation, or QTc prolongation when compared to a placebo in patients with underlying cardiovascular disease.

Bonnett commented on a previous article (13) about the more attention needed in prescribing SSRIs during COVID-19 adding interesting discussion points. In particular, in the light of the increased SSRIs prescription trend in coronavirus infections (i.e., fluvoxamine) it should also be considered the potential occurrence of an anxiety/jitteriness syndrome (AJS, also known as “activation syndrome”) as a common ADR of SSRIs. Therefore, the AJS might be mistakenly identified as neuropsychiatric or gastrointestinal symptoms of COVID-19 or as a worsening of an existing COVID-19 condition.

The team led by Xu et al. conducted a systematic review between the citalopram plasma concentration and treatment outcomes in depression. The review did not establish a clear correlation between citalopram plasma concentration and clinical or cost-related outcomes, but there was some limited evidence hinting at improved treatment effectiveness in patients with plasma concentrations above 50 or 53 ng/mL.

Xiang et al. aimed to examine the impact of a Chinese policy implemented on September 1, 2019, which classified oxycodone/acetaminophen as a psychotropic medicine due to its history of misuse, with the goal to regulate its use in medical institutions. Through a prescription data analysis from five tertiary hospitals in Xi'an city over 42 months, authors concluded that the stricter regulation of oxycodone/acetaminophen helped reduce the risk of misuse among short-term drug users.

In another retrospective analysis run in a long-term hospital with chronic schizophrenia patients with co-existing type 2 diabetes who were prescribed olanzapine or risperidone as primary antipsychotic medication with or without co-prescribing aripiprazole (Liu et al.). Liu et al. compared various parameters between the two groups and assessed the factors influencing prolactin levels. The results showed that the co-aripirazole group had significantly higher levels of fasting blood glucose, blood uric acid, cholesterol, and triglycerides, but there was no difference in prolactin levels between the two groups.

An EUDRAVigilance dataset screening led by Cicala et al. aimed to provide a more comprehensive understanding of the safety and tolerability of paliperidone palmitate (PP) using real-world pharmacovigilance data. This analysis of 8,152 reports suggested that the safety and tolerability profiles of PP are generally consistent with other second generation long- acting anitpychotics, with only higher probabilities of reporting specific ADRs in PP-related reports, particularly related to sexual dysfunctions, haemodynamic edema, effusions, fluid overload, and fertility disorders.

According to a nationwide retrospective study between 1998 and 2019 in Australia conducted by Alsaleh et al. potential errors in the administration of psychotropic drugs are a common cause of hospitalization in Australia, often requiring overnight stays. The concerning prevalence of such errors among individuals aged 20–39 years underscores the need for further investigation and research to understand the risk factors associated with hospitalization due to psychotropic drug administration errors.

Hamasaki and Yanai conducted a retrospective cohort study with the aim to investigate the impact of psychotropic drug use on handgrip strength and hospitalization in individuals with type 2 diabetes. Authors concluded that the use of psychotropic drugs may increase the likelihood of repeated hospitalizations among patients with type 2 diabetes. Morevoer, the study suggests that skeletal muscle strength, as reflected by handgrip strength, may have a role in reducing the risk of hospitalization in individuals receiving psychotropic drug treatment.

The study by Joung investigated the gender differences in ADRs associated with zolpidem, with data from the Korea voluntary adverse drug events reporting system (KAERS) for the years 2015–2019. Of all the ADRs with gender differences in reporting risk, somnambulism stood out as the most consistent and substantial difference. This suggests that women may have a higher susceptibility to somnambulism, which is a potentially serious adverse effect associated with zolpidem.

While psychopharmacological research has already generated a substantial amount of data on numerous facets, we contend that the clinical perspectives presented in these articles offer a practical and innovative viewpoint that could lead to a more precise allocation of resources, piquing the interest of both clinicians and researchers (14, 15). In light of the psychopharmacological trends discussed in the collected papers, it is crucial to underscore the pressing need for a deeper exploration of ADRs associated with psychotropic medications. The findings compiled in this Research Topic do not discourage the use of psychotropic drugs but rather advocate for their thoughtful and informed prescription.

## Author contributions

RF: Conceptualization, Data curation, Writing – original draft. MSo: Conceptualization, Writing – review & editing. MSh: Conceptualization, Writing – review & editing.
